# Comprehensive Analysis of SARS-CoV-2 Dynamics in Bangladesh: Infection Trends and Variants (2020–2023)

**DOI:** 10.3390/v16081263

**Published:** 2024-08-07

**Authors:** Mst. Noorjahan Begum, Selim Reza Tony, Mohammad Jubair, Md. Shaheen Alam, Yeasir Karim, Mohammad Hridoy Patwary, Sezanur Rahman, Mohammad Tanbir Habib, Anisuddin Ahmed, Mohammad Enayet Hossain, Mohammed Ziaur Rahman, Manjur Hossain Khan, Tahmina Shirin, Firdausi Qadri, Mustafizur Rahman

**Affiliations:** 1 Infectious Diseases Division, International Centre for Diarrhoeal Disease Research, Bangladesh (icddr,b), Mohakhali, Dhaka 1212, Bangladesh; noorjahan.maliha@icddrb.org (M.N.B.); selim.tony@icddrb.org (S.R.T.); mohammad.jubair@icddrb.org (M.J.); shaheen.alam@icddrb.org (M.S.A.); yeasir.karim@icddrb.org (Y.K.); sezanur.rahman@icddrb.org (S.R.); enayet.hossain@icddrb.org (M.E.H.); mzrahman@icddrb.org (M.Z.R.); fqadri@icddrb.org (F.Q.); 2 Maternal and Child Health Division, International Centre for Diarrhoeal Disease Research, Bangladesh (icddr,b), Mohakhali, Dhaka 1212, Bangladesh; mpatwary@isrt.ac.bd (M.H.P.); anisuddin@icddrb.org (A.A.); 3 Institute for Developing Science and Health Initiatives, Dhaka 1206, Bangladesh; tanbir.habib@ideshi.org; 4 Institute of Epidemiology, Disease Control and Research (IEDCR), Dhaka 1212, Bangladesh; khanmanjur56@gmail.com (M.H.K.); tahmina.shirin14@gmail.com (T.S.)

**Keywords:** COVID-19, SARS-CoV-2 variants, risk difference, Bangladesh

## Abstract

The first case of COVID-19 was detected in Bangladesh on 8 March 2020. Since then, the Government of Bangladesh (GoB) has implemented various measures to limit the transmission of COVID-19, including widespread testing facilities across the nation through a laboratory network for COVID-19 molecular testing. This study aimed to analyze the dynamics of SARS-CoV-2 in Bangladesh by conducting COVID-19 testing and genomic surveillance of the virus variants throughout the pandemic. Nasopharyngeal swabs were collected from authorized GoB collection centers between April 2020 and June 2023. The viral RNA was extracted and subjected to real-time PCR analysis in icddr,b’s Virology laboratory. A subset of positive samples underwent whole-genome sequencing to track the evolutionary footprint of SARS-CoV-2 variants. We tested 149,270 suspected COVID-19 cases from Dhaka (n = 81,782) and other districts (n = 67,488). Of these, 63% were male. The highest positivity rate, 27%, was found in the >60 years age group, followed by 26%, 51–60 years, 25% in 41–50 years, and the lowest, 9% in under five children. Notably, the sequencing of 2742 SARS-CoV-2 genomes displayed a pattern of globally circulating variants, Alpha, Beta, Delta, and Omicron, successively replacing each other over time and causing peaks of COVID-19 infection. Regarding the risk of SARS-CoV-2 infection, it was observed that the positivity rate increased with age compared to the under-5 age group in 2020 and 2021. However, these trends did not remain consistent in 2022, where older age groups, particularly those over 60, had a lower positivity rate compared to other age groups due to vaccination. The study findings generated data on the real-time circulation of different SARS-CoV-2 variants and the upsurge of COVID-19 cases in Bangladesh, which impacted identifying hotspots and restricting the virus from further transmission. Even though there is currently a low circulation of SARS-CoV-2 in Bangladesh, similar approaches of genomic surveillance remain essential for monitoring the emergence of new SARS-CoV-2 variants or other potential pathogens that could lead to future pandemics.

## 1. Introduction

The severe acute respiratory syndrome coronavirus 2 (SARS-CoV-2) virus has instigated a global pandemic, leading to approximately 7 million fatalities worldwide as of 13 April 2024 [1]. Bangladesh’s first case of COVID-19 was identified on 8 March 2020. Subsequently, as new variants of the SARS-CoV-2 virus emerged, the country encountered multiple waves of COVID-19 infections, resulting in a cumulative total of over 20 million cases and 29,498 deaths to date [2]. The impact of the virus has been substantial across various sectors in Bangladesh, particularly affecting the economy and education.

In response to the COVID-19 pandemic, the Government of Bangladesh (GoB), guided by the Director General of Health Services (DGHS) under the Ministry of Health and Family Welfare (MoHFW), enacted several preventive measures. These included enforcing physical distancing, mandating mask-wearing, promoting frequent handwashing, and implementing strict lockdowns to curb the virus’s spread. Moreover, nationwide testing facilities were established, and icddr,b was incorporated into the laboratory network for COVID-19 molecular testing. The Virology Laboratory at icddr,b, led by a team of scientists and researchers, conducted extensive investigations into SARS-CoV-2.

Throughout the period spanning from April 2020 to June 2023, a group of studies have been conducted in Bangladesh focused on COVID-19 testing [3,4,5]. Scientists also remained vigilant concerning the emergence of SARS-CoV-2 variants. The genetic evolution inherent to RNA viruses like SARS-CoV-2 contributed to the emergence of new variants over time. Several variants of concern (VOC), characterized by their increased transmissibility, emerged, including Alpha (B.1.1.7), Beta (B.1.351), Gamma (P.1), Delta (B.1.617.2), and Omicron (B.1.1.529), which have spread globally, including within Bangladesh [6,7,8]. The emergence of these variants significantly contributed to the successive waves of COVID-19 epidemics across the globe [9].

The global containment strategies for SARS-CoV-2 have varied significantly, reflecting differing public health policies, healthcare capacities, and socio-economic conditions. Initial strategies ranged from strict lockdowns and widespread testing, as seen in New Zealand, to more relaxed approaches emphasizing herd immunity, as initially adopted by Sweden [10]. Studies have shown that stringent early interventions, such as those implemented in China, helped to significantly reduce transmission rates [11]. Conversely, countries with delayed responses experienced higher infection rates and mortality [12]. Seasonality has also influenced COVID-19 dynamics, with some studies suggesting a higher transmission rate in colder, less humid conditions [13]. Additionally, the emergence of variants, such as the Delta and Omicron, has further complicated containment efforts due to their increased transmissibility and partial resistance to existing vaccines [14,15]. These factors highlight the complex interplay between public health interventions, environmental conditions, and viral evolution in managing the COVID-19 pandemic.

In the early phase of the pandemic, the rapid spread of SARS-CoV-2 and the limited knowledge of its pathogenicity and vaccine efficacy, it became imperative to conduct extensive testing to identify confirmed COVID-19 cases and subsequently isolate them. At present, despite the low prevalence of COVID-19-positive cases within the country, the significance of testing persists, particularly regarding vulnerable populations. Within the broader framework of public health mitigation efforts, testing plays a pivotal role in characterizing disease prevalence, transmission dynamics, and contagiousness. Moreover, the potential resurgence of novel variants perpetually threatens public health. Consequently, the continuous monitoring of SARS-CoV-2 variants remains a critical imperative. 

The study’s objective was to explore the distribution of COVID-19 cases over age and sex and the seasonality patterns of SARS-CoV-2 over the past three years in Bangladesh. The insights gathered from this study are poised to play an instrumental role in aiding policymakers in making timely decisions and supporting researchers in their ongoing efforts to develop and periodically update effective vaccine candidates and therapeutic strategies.

## 2. Materials and Methods

### 2.1. Ethics Approval and Participant Consent

The study protocol (PR-21065) received approval from the Institutional Review Board of icddr,b (www.icddrb.org). During the COVID-19 pandemic, the government designated particular booths for collecting samples and data from COVID-19 suspected cases who were enrolled in our study. Due to the challenges in obtaining written consent during this period, participants were only required to give verbal consent. The data and sample records were kept under a code number rather than participants’ names, and the data were analyzed anonymously.

### 2.2. Inclusion Criteria

The study encompassed individuals of all age groups and genders suspected of COVID-19, as recommended by the World Health Organization (WHO).

### 2.3. Study Sites and Population

**Figure 1:**
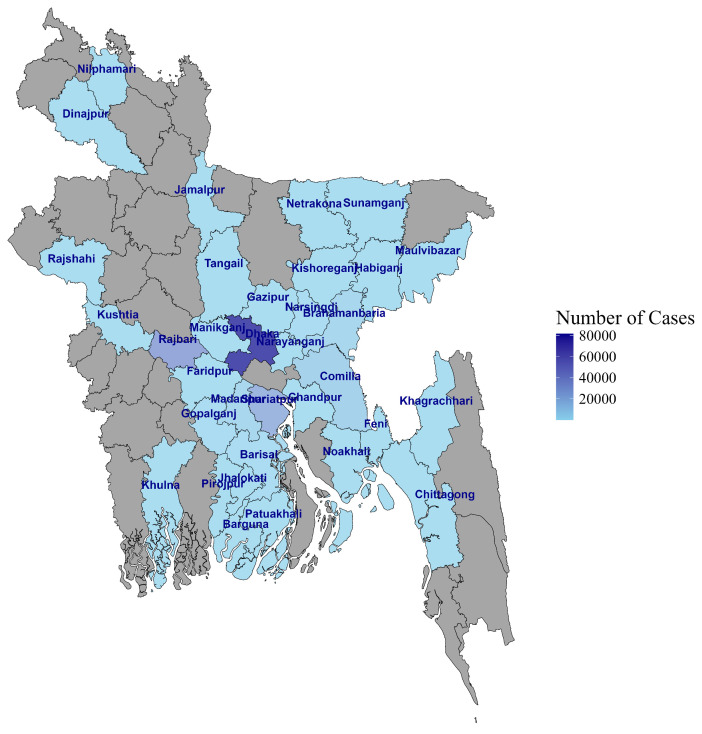
Map of Bangladesh showing the sample collection sites.

Between 4 April 2020 and 25 June 2023, COVID-19-suspected individuals who visited government-assigned COVID-19 sample collection booths in Dhaka and district-level hospitals outside Dhaka were recruited for this study (Figure 1). Nasopharyngeal (NP) swab samples and metadata were collected and transported to the Virology Laboratory at icddr,b for testing and reporting to the GoB. The data were accessed for research following IRB approval on 16 August 2021.

### 2.4. Specimen Collection

NP samples were obtained from all participants and stored in a 2 mL viral transport medium (VTM). For 1 L of VTM, the composition includes 1 packet of DMEM (Dulbecco’s Modified Eagle Medium) powder, 2% HEPES (4-(2-Hydroxyethyl) piperazine-1-ethanesulfonic acid), 1% L-Glutamine (200 mM), 1% Sodium Pyruvate (100 mM), 3.7 g Sodium Bicarbonate, 1% Penicillin-Streptomycin (10,000 IU/mL), 0.8% Fungizone (250 µg/mL), and 2.5% Bovine Serum Albumin (7.5% solution). These VTM tubes, containing the swab stick, were then placed in a cooler box with an ice pack (maintaining temperatures of 2–8 °C) or a nitrogen tank for sample transportation from collection sites (Dhaka and hospitals outside Dhaka) to the Virology Laboratory of icddr,b.

### 2.5. Sample Processing, Viral Nucleic Acid Extraction and Real-Time RT-qPCR

NP samples were processed within a dedicated BSL-2 laboratory, adhering to BSL-3 practices, all under the containment of a certified Class II biological safety cabinet (BSC). Viral nucleic acid (RNA) was extracted and purified from samples using either an automated extractor (KingFisher Flex96 system) with an Invimag Pathogen kit or manually with a QiaAmp-viral mini kit following the manufacturer’s guidelines. At first, NP swab samples were treated with lysis buffer (AVL-Guanidine thiocyanate), followed by washing and elution steps of the extraction procedure.

After extraction, the SARS-CoV-2 viral RNA underwent real-time RT-qPCR assay. We targeted the nucleocapsid protein (N gene) and RNA-dependent RNA polymerase (RdRp gene) to detect SARS-CoV-2 RNA. For the RdRp gene, the forward primer, reverse primer, and fluorescent probe sequences were CCCTGTGGGTTTTACACTTAA, ACGATTGTGCATCAGCTGA, and 5′-FAM-CCGTCTGCGGTATGTGGAAAGGTTATGG-BHQ1-3′, respectively. For the N gene, the forward primer, reverse primer, and fluorescent probe sequences were GGGGAACTTCTCCTGCTAGAAT, CAGACATTTTGCTCTCAAGCTG, and 5′-FAM-TTGCTGCTGCTTGACAGATT-TAMRA-3′, respectively. The assay utilizes AgPath-ID™ One-Step RT-PCR Reagents (Thermo Fisher Scientific Inc., Waltham, MA, USA) and was performed on the CFX96 Touch™ Real-time PCR Detection System (Bio-Rad Laboratories, Inc., Hercules, CA, USA). The reaction setup includes a total volume of 25 μL, comprising 5 μL of viral RNA (25 ng/μL), 12.5 μL of 2X RT-PCR buffer, 1 μL (200 U/μL) of 25X RT-PCR enzyme mix, 0.5 μL of forward and reverse primers (0.8 pmol each), and 0.5 μL of probes (0.2 pmol each). The thermal cycling conditions involve reverse transcription at 45 °C for 10 min, RT inactivation/initial denaturation at 95 °C for 10 min, followed by 45 amplification cycles of 95 °C for 10 s and 60 °C for 45 s. The PCR data are analyzed using CFX Manager Software version 2.2, and a Ct value < 37 was regarded as confirmed COVID-19 positive [16,17,18].

### 2.6. SARS-CoV-2 Genome Sequencing

Initially, our approach encompassed using Sanger sequencing to pinpoint variants within the spike protein region. After this, we processed the viral RNA for sequencing using the Next Generation Sequencing (NGS) platforms. We used Illumina MiSeq and Oxford Nanopore MinION technologies for sequencing. The Illumina-based sequencing was conducted using the Illumina COVIDseq^TM^ assay (20051273) for library preparation. The process involved the preparation of indexed libraries, which were subsequently pooled together. These pooled libraries underwent sequencing for 300 cycles using the MiSeq V3 2 × 300 cycle (MS-102-3003) cartridge. For samples sequenced using the Oxford Nanopore MinION technology, viral RNA was first converted into cDNA using the Lunascript RT SuperMix Kit (NEB, E3010). Following cDNA synthesis, the ARCTIC protocol was utilized to amplify viral genomic regions, which are critical for effective surveillance. The amplified viral amplicons were prepared for sequencing using the Native Barcoding Kit (EXP-NBD104). The prepared libraries were then sequenced on R9 flow cells for 8 h, facilitating real-time data acquisition and analysis.

### 2.7. Data Analysis

The statistical analysis was performed using SPSS 17.0, and the graphs were generated using R (V 4.3.2). This study expressed continuous variables as means with 95% confidence intervals, while categorical variables were presented as percentages. The probability of testing positive was modeled using an adjusted linear probability model. Also, the difference in positivity rate for males and females was tested for statistical significance using a two-sample Welch *t*-test. The trend in risk of being positive over the years for different subgroups was tested for significance using the Mann–Kendall trend test. 

Furthermore, we analyzed the sequencing data using DRAGEN COVID Lineage for Illumina-generated sequences and EPI2ME pipeline for Oxford Nanopore sequences to obtain high-quality data. The analysis also includes identifying variants of concern (VOCs) and monitoring the spread of these variants in the population. Moreover, viral genomics data underwent analysis through Nextclade https://clades.nextstrain.org (accessed on 4 July 2023) to compare our sequences to the SARS-CoV-2 reference genome and assign them to clades. We uploaded the generated data to the Global Initiative on Sharing All Influenza Data (GISAID) database, which is the primary repository for SARS-CoV-2 sequences. 

## 3. Results

### 3.1. Demographic Characteristics of the Study Participants

Between April 2020 and June 2023, 149,270 suspected COVID-19 cases through GoB platforms underwent testing at the Virology laboratory. Of these cases, 81,782 were from Dhaka, while the remaining were from areas outside Dhaka. Regarding gender distribution, approximately 63% of the subjects were male (Table 1). Among the samples collected in Dhaka, 54% were from males and 46% from females. The overall mean age of the study participants was 36 years. The mean was slightly lower (34 years) in participants within Dhaka compared to those outside Dhaka (38 years). When considering age groups, overall, the lowest number of samples (n = 2138) were collected from individuals under the age of 5 years, followed by those aged >5 to 17 years (n = 10,037) and over 60 years (n = 10,431). On the contrary, the highest number (n = 53,002) of samples were obtained from the 18–30 years age group, followed by 31–40 years (n = 36,893), 41–50 years (n = 21,686), and 51–60 years group (n = 15,083). Taking location into account, 1406 (<5 years), 5499 (5–17 years), 31,588 (18–30 years), 20,309 (31–40 years), and 11,234 (41–50 years) individuals submitted their samples from Dhaka. In contrast, the number of samples from outside Dhaka was higher for the older age groups, with 7874 samples from individuals aged 51–60 and 5894 from those over 60 years old, compared to 7209 and 4537 samples, respectively, from Dhaka.

### 3.2. Yearly Patterns of COVID-19 Cases and Infection Risk by SARS-CoV-2

Of all the participants (149,270), 33,090 individuals tested positive, resulting in an overall positivity rate of 22%. The yearly distribution of COVID-19 positivity rates is shown in Table 2. Overall, the positivity rate was lower in Dhaka (21%) compared to other districts (23%). Female participants were observed to have a significantly higher (*p*-value < 0.001) positivity rate (23%) compared to their male counterparts (22%). Moreover, over the years we found a significant positivity rate among different age groups. 

Overall, for children under 5 years old, the positivity rate was relatively low at 9%. As we move to the 5–17 years age group, the positivity rate increases to 16%, showing a notable rise in positive cases. In the young adult group, aged 18–30, the positivity rate increased to 20%. For individuals aged 31–40, the positivity rate continues to rise, reaching 23%, while the 41–50 age group has an even higher rate of 25%. The positivity rate peaks in the older age groups, with the 51–60 years group at 26% and those over 60 years old with the highest rate at 27%. These data highlight a trend where the positivity rate generally increases with age, indicating that older age groups have higher proportions of positive COVID-19 cases (Table 2).

Moreover, the risk difference (RD) of being COVID-19-positive was modeled for 2020, 2021, and 2022 (Table 2). In 2020, males had more chances of being positive (Risk difference, RD) than females. This direction changed in the upcoming years (2021, 2022), and females were observed to have a higher risk of being positive. Overall, males had a significantly lower positivity rate compared to females.

Regarding age groups, it was observed in 2020 and 2021 that the positivity rate for SARS-CoV-2 increased with age compared to the under-5 age group (reference group). During these years, older individuals were at the highest risk of testing positive for the virus, with increased rates of 15% for the 41–50 age group, 17% for the 51–60 age group, and 18% for those over 60 years old, compared to the under-5 group. However, these trends did not remain consistent in 2022. During that year, older age groups, particularly those over 60, had a lower increased positivity rate than other age groups. 

Overall, living outside Dhaka was associated with a 2% positivity rate increase compared to Dhaka. As there were very few positive cases (n = 12) in 2023, we did not perform any modeling for 2023. 

### 3.3. Distribution of COVID-19 Positivity over Age, Sex, and Locations

**Figure 2:**
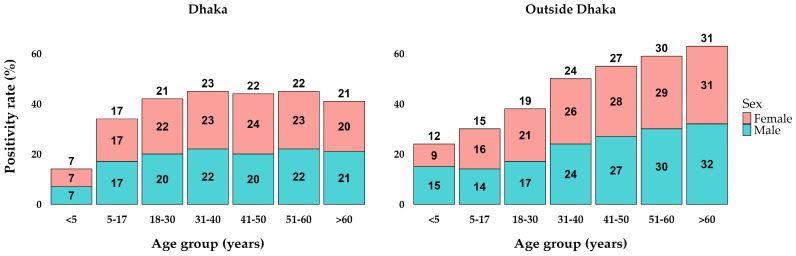
Percentages of positive cases in age and sex groups between locations.

The analysis of positivity rates by age and sex groups in Dhaka and outside Dhaka reveals notable trends. In Dhaka, the positivity rates start at 7% in the <5 years age group, increase to 17% in the 5–17 years age group, and reach their peak at 23% in the 31–40 years age group. Subsequently, the rates stabilize around 22% for the 41–50 and 51–60 years age groups, before slightly decreasing to 21% in those over 60 years old. The rates were relatively similar between sexes within each age group, showing minimal differences (Figure 2). Outside Dhaka, the positivity rates were consistently higher across all age groups compared to Dhaka. The rates begin at 12% for the <5 years age group, rise to 15% in the 5–17 years age group, and continue to increase, reaching 19% in the 18–30 years age group. The positivity rate peaks at 31% in those over 60 years old. Both locations exhibit an upward trend in positivity rates with increasing age, with outside Dhaka showing a more pronounced rise. 

### 3.4. Seasonal Patterns of SARS-CoV-2 and Their Circulating Variants

**Figure 3:**
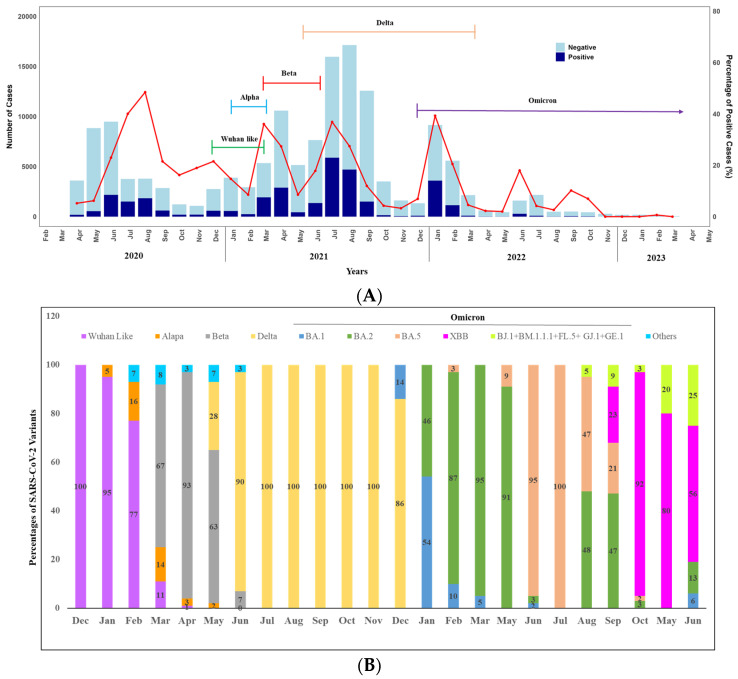
(**A**) Monthly distribution of COVID-19 cases in Bangladesh: April 2020–June 2023 (Bar diagram) and SARS-CoV-2 variants: December 2020–June 2023 (Closed line), Seasonal pattern (line graph). (**B**) Seasonal distribution of SARS-CoV-2 variants over the years (2020–2023). Other Include Nigeria (B.1.525), VOC (B.1.1.7 + E484K), and VOI-5 (N394K + E484K + N501Y).

We observed the seasonality of SARS-CoV-2 over the year by analyzing the positivity rate (Figure 3A,B). A total of 188 positive cases were detected in April and 549 in May. The case numbers began to rise, reaching the highest peak in June (n = 2181), followed by August (n = 1841) and July (n = 1510) (Figure 3A). A notable decrease was observed in September, with 615 cases detected. In October, November, and December, the numbers stood at 199, 205, and 593 cases, respectively. Overall, in 2020, we found a peak in the COVID-19 positivity rate between June to August. Notably, only virus sequencing was conducted in December 2020, and it was found that 100% of the circulated variants were the original strain of SARS-CoV-2. 

However, we observed two peaks in 2021 between March to May and June to September. In 2021, the highest peak of COVID-19 cases occurred in July (5893), followed by August (4706). There was a decline in September with 1507 cases, further reducing to 150 in October, 53 in November, and 93 in December. Throughout 2021, we engaged in monthly sequencing of positive cases to monitor the variants. The alpha variant was detected in the first week of January 2021 (5% only). It remained in circulation until mid-March (16% in February and 14% in March) alongside the original strain.

A significant shift in variant distribution was observed after the emergence of the Beta variant on 16 March 2021. The Beta variant quickly became the prevailing variant, replacing most other variants. Notably, by April 2021, the Beta variant constituted approximately 95% of the circulating variants in Dhaka, although several Delta variants were also detected in May 2021. In June 2021, the Delta variant (90%) surpassed the Beta variant and remained dominant (100%) throughout the year until the Omicron variant emerged at the end of December.

Moving on to 2022, we observed two peaks. The highest peak was observed in January (n = 3600), followed by February (n = 1148). Positivity rates were significantly lower in the other months, except in June, when 289 positive cases were reported (Figure 3A). In terms of variant analysis, the Omicron variant was circulated throughout the year with various lineages. In February BA.1 and BA.2 were in circulation with 54% and 46%, respectively. From March to May, BA.2 was dominantly (more than 90%) circulated. However, the positivity rate was lowest in 2023 over the years, and at the end of May 2023, XBB emerged, and all the variants identified following the month were XBB with different lineages, including BJ.1 + BM.1.1.1 + FL.5 + GJ.1 + GE.1 (Figure 3B). 

Moreover, when we analyzed the data considering seasonality and location, we did not find any difference.

## 4. Discussion

This is the first report from Bangladesh that covers three years of the COVID-19 pandemic, with 149,270 participants tested in the Virology laboratory, icddr,b. This study provided an in-depth analysis of the SARS-CoV-2 infection dynamics, the seasonal distribution of variants and the assessment of the risk of the disease in different age and sex groups. 

Analyzing age-related infection patterns revealed that the lowest positivity rate was observed in children and adolescents below 18 years, while the highest positivity was noted among older participants. These results aligned with findings from previous studies [19,20,21,22,23]. The differences in positivity in different age groups could be attributed to various factors, including the co-morbid conditions, immunity, and behavioral practices of varying age groups [19]. Interestingly, an intriguing observation was made regarding the risk posed to individuals over the age of 50 in 2022, showing a decrease in their susceptibility compared to their heightened risk during the initial virus emergence in 2020. This risk reduction suggests potential protection might be due to natural infections and the priority vaccination of older adults in Bangladesh [24]. Different global studies conducted in various populations showed that the immune response induced by natural infection or vaccination could be protective for further SARS-CoV-2 infection [25,26,27]. Supporting these findings, a study demonstrated a notable decrease in COVID-19 positivity rates in countries with high pre-vaccination levels [28]. Furthermore, the study highlighted that while COVID-19 vaccines may offer limited protection against infection, they are highly effective in lessening the disease severity, therefore significantly reducing the chances of hospitalizations and ICU admissions [29].

Regarding gender-related infection rates, the study found that the overall positivity rates were almost similar between males and females. This finding diverged from other studies [30,31,32,33], potentially due to variations in the male-to-female ratio among study participants (the number of male participants was higher than that of females). For instance, studies with more balanced gender ratios exhibited different outcomes [30,31]. Bangladesh-specific cultural context, with certain conservative practices and gender-specific exposures, might contribute to the observed differences of less female participation, underscoring the importance of addressing gender disparities in testing and promoting awareness.

The geographical variation in infection rates highlights that the overall infection rate was lower in Dhaka compared to other districts, likely due to better healthcare infrastructure, higher awareness, and more stringent public health measures. 

Regarding the seasonality of SARS-CoV-2 infection over the pandemic years, we did not find any consistent pattern. However, newly emerged variants played a vital role in the four distinct waves of Bangladesh [34]. The first wave occurred in March 2020, followed by intermittent peaks till August 2020, driven primarily by the original virus strain [6,34,35]. The second wave, beginning in March 2021 with the emergence of Alpha, showed peaks in March and April, co-circulated with the prevalence of the Beta variant [6,34,36]. Subsequently, the third wave surged from June 2021 onward, driven by the Delta variant. The fourth wave, induced by the Omicron variant, emerged in December 2021, with sharp spikes in cases and still circulating at a lower rate [34]. The study proposed that the emergence and predominance of new variants contributed to the occurrence of waves, as these variants displaced each other over time. 

Notably, the decline in infection rates from October 2022 could be attributed to the substantial vaccination coverage achieved in Bangladesh since February 2021. The observed shift from a pandemic to an endemic phase was linked to Omicron variants, possibly interacting with natural immunity and vaccination efforts [37]. While vaccines have proven effective in reducing the severity of illness, hospitalization rates, and mortality, they also exert selective pressure on the virus, potentially accelerating the emergence of new variants. Another study highlighted that regions with high vaccination coverage experienced a notable shift in the prevalence of certain variants. For instance, the Omicron variant, which emerged in late 2021, displayed significant mutations in its spike protein, enabling it to partially evade the immune protection conferred by vaccines [38]. Furthermore, another comprehensive analysis demonstrated that while vaccines effectively reduce the transmission of earlier variants, the emergence of newer variants like Delta and Omicron underscores the necessity for continuous monitoring and potential updates to vaccine formulations to maintain efficacy [39].

Moreover, vaccine-induced immunity, combined with natural infection, has led to a complex interplay influencing the viral evolution landscape. Studies suggest that hybrid immunity—stemming from both vaccination and previous infection—provides robust protection against severe disease but may not completely prevent the transmission of emerging variants [40]. Thus, continuous variant monitoring remains vital to predict and manage new waves and to facilitate vaccine adaptation.

The important findings of this research identified significant age-related differences in COVID-19 positivity rates and highlighted the impact of vaccination and emerging variants on infection dynamics. Specifically, additional research can explore age-specific immune responses to better understand why children and adolescents have lower positivity rates and why older adults saw a reduced risk over time. Moreover, examining the long-term efficacy of vaccines and the role of natural infections can help optimize vaccination strategies. Continuous monitoring of new variants is crucial to adapt public health measures and vaccine formulations, ensuring they remain effective against evolving strains of the virus. These studies will provide critical insights for tailoring public health strategies, improving vaccine deployment, and ultimately managing future outbreaks more effectively.

This study provides essential insights into SARS-CoV-2 infection dynamics in Bangladesh over three years, highlighting age-related infection patterns, gender and regional differences, and the impact of variants. Continuous monitoring of new variants is crucial to monitor future potential pandemic threats as well as update vaccine candidate selection. Further research is needed to adapt public health strategies, optimize vaccination efforts, and manage future outbreaks effectively.

## 5. Conclusions

In conclusion, our real-time sample and data collection, analysis, and reporting to the GoB database were crucial in quickly identifying COVID-19 outbreaks, emerging trends, and hotspots. This information was effectively utilized in public health interventions to curb further virus transmission. Although the country currently reports relatively low numbers of COVID-19 cases, maintaining similar genomic surveillance approaches is essential for monitoring the emergence of new SARS-CoV-2 strains or other potential pathogens that could lead to future pandemics.

## Figures and Tables

**Table 1:** Demographic characteristics of the study participants.

Category	Dhaka	Outside Dhaka	Total
**Suspected COVID-19 cases**	81,782 (54.8)	67,488 (45.2)	149,270 (100)
**Sex**			
**Male**	50,406 (54.0)	43,022 (46.0)	93,428 (62.6)
**Female**	31,376 (56.2)	24,466 (43.8)	55,842 (37.4)
**Age (years)**			
**<5**	1406 (65.8)	732 (34.2)	2138 (1.4)
**5–17**	5499 (54.8)	4538 (45.2)	10,037 (6.7)
**18–30**	31,588 (59.6)	21,414 (40.4)	53,002 (35.5)
**31–40**	20,309 (55.0)	16,584 (45.0)	36,893 (24.7)
**41–50**	11,234 (51.8)	10,452 (48.2)	21,686 (14.5)
**51–60**	7209 (47.8)	7874 (52.2)	15,083 (10.1)
**>60**	4537 (43.5)	5894 (56.5)	10,431 (6.9)
**Mean (SD)**	34.2 (14.9)	37.8 (16.5)	35.8 (15.7)

Data presented as n (%).

**Table 2:** COVID-19 positivity and Risk Difference of SARS-CoV-2 infections.

Categories	2020			2021			2022			Overall		
	PR (%)		RD [*p*-Value]	PR (%)		RD [*p*-Value]	PR (%)		RD [*p*-Value]	PR (%)		RD [*p*-Value]
Age group (Years)												
<5	10.6	*p* < 0.001	Ref	8.8	*p* < 0.001	Ref	8.4	*p* < 0.001	Ref	9.1	*p* < 0.001	Ref
5–17	13.7		0.03 [0.13]	17.6		0.08 [5.56 × 10^−9^]	16.6		0.09 [3.30 × 10^−6^]	16.4		0.07 [5.84 × 10^−13^]
18–30	16.1		0.05 [0.006]	20.7		0.12 [1.01 × 10^−18^]	23.1		0.15 [1.21 × 10^−20^]	20.0		0.11 [9.39 × 10^−34^]
31–40	22.2		0.11 [6.98 × 10^−9^]	23.4		0.15 [1.58 × 10^−26^]	25.5		0.18 [1.96 × 10^−26^]	23.4		0.14 [4.16 × 10^−55^]
41–50	26.1		0.15 [1.59 × 10^−14^]	24.0		0.15 [1.60 × 10^−26^]	23.4		0.16 [2.90 × 10^−19^]	24.5		0.15 [3.83 × 10^−60^]
51–60	27.8		0.17 [4.46 × 10^−17^]	26.5		0.17 [3.74 × 10^−33^]	22.7		0.15 [5.05 × 10^−17^]	26.3		0.17 [1.86 × 10^−71^]
>60	28.9		0.18 [5.77 × 10^−18^]	27.7		0.18 [1.34 × 10^−34^]	19.5		0.13 [9.26 × 10^−11^]	26.8		0.18 [5.76 × 10^−71^]
Sex												
Female	19.6	*p* < 0.001	Ref	24.0	*p* < 0.001	Ref	22.9	*p* = 0.03	Ref	22.9	*p* < 0.001	Ref
Male	21.7		0.01 [0.01]	21.7		−0.02 [1.45 × 10^−14^]	22.3		−0.01 [0.09]	21.8		−0.02 [3.61 × 10^−12^]
Location												
Dhaka	21.6	*p* = 0.06	Ref	20.3	*p* < 0.001	Ref	23.4	*p* < 0.001	Ref	21.1	*p* < 0.001	Ref
Outside Dhaka	21.0		−0.01 [0.04]	27.5		0.07 [5.07 × 10^−103^]	17.2		−0.06 [1.44 × 10^−16^]	23.6		0.02 [4.40 × 10^−22^]
Intercept for RD	0.12 [6.03 × 10^−7^]			0.02 [1.33 × 10^−9^]			0.15 [1.37 × 10^−8^]			0.07 [2.31 × 10^−23^]		

PR—Positivity rate, RD—Risk Difference, LPM—Linear Probability Model.

## Data Availability

The authors confirm that the data supporting the findings of this study are available within the article; additional data related to this study are available from the corresponding author upon reasonable request.

## References

[1] Worldometer (2024). COVID-19 CORONAVIRUS PANDEMIC. https://www.worldometers.info/coronavirus/.

[2] Director General of Health Services (DGHS) (2024). COVID-19 Dynamic Dashboard for Bangladesh. https://dashboard.dghs.gov.bd/pages/covid19.php.

[3] Kawser Z., Hossain M., Suliman S., Lockman S., Gitaka J., Bandawe G., Rahmat R., Hasan I., Siddik A.B., Afrad M.H. (2022). An assessment of a rapid SARS-CoV-2 antigen test in Bangladesh. Am. J. Trop. Med. Hyg..

[4] Maniruzzaman M., Islam M.M., Ali M.H., Mukerjee N., Maitra S., Kamal M.A., Ghosh A., Castrosanto M.A., Alexiou A., Ashraf G.M. (2022). COVID-19 diagnostic methods in developing countries. Environ. Sci. Pollut. Res..

[5] Sazed S.A., Kibria M.G., Zamil M.F., Hossain M.S., Khan J.Z., Juthi R.T., Hossain M.E., Ahmed D., Noor Z., Haque R. (2022). Direct Nasal Swab for Rapid Test and Saliva as an Alternative Biological Sample for RT-PCR in COVID-19 Diagnosis. Microbiol. Spectr..

[6] Afrin S.Z., Islam M.T., Paul S.K., Kobayashi N., Parvin R. (2022). Dynamics of SARS-CoV-2 variants of concern (VOC) in Bangladesh during the first half of 2021. Virology.

[7] Dhama K., Nainu F., Frediansyah A., Yatoo M.I., Mohapatra R.K., Chakraborty S., Zhou H., Islam M.R., Mamada S.S., Kusuma H.I. (2023). Global emerging Omicron variant of SARS-CoV-2: Impacts, challenges and strategies. J. Infect. Public Health.

[8] Rahman M.S., Hoque M.N., Chowdhury S.R., Siddique M.M., Islam O.K., Galib S.M., Islam M.T., Hossain M.A. (2023). Temporal dynamics and fatality of SARS-CoV-2 variants in Bangladesh. Health Sci. Rep..

[9] Aleem A., Akbar Samad A.B., Vaqar S. (2024). Emerging Variants of SARS-CoV-2 and Novel Therapeutics against Coronavirus (COVID-19). StatPearls [Internet].

[10] Paterlini M. (2020). Closing borders is ridiculous’: The epidemiologist behind Sweden’s controversial coronavirus strategy. Nature.

[11] Tian H., Liu Y., Li Y., Wu C.H., Chen B., Kraemer M.U., Li B., Cai J., Xu B., Yang Q. (2020). An investigation of transmission control measures during the first 50 days of the COVID-19 epidemic in China. Science.

[12] Baker M.G., Kvalsvig A., Verrall A.J., Telfar Barnard L., Wilson N. (2020). New Zealand’s elimination strategy for the COVID-19 pandemic and what is required to make it work. N. Z. Med. J..

[13] Neher R.A., Dyrdak R., Druelle V., Hodcroft E.B., Albert J. (2020). Impact of seasonal forcing on a potential SARS-CoV-2 pandemic. medRxiv.

[14] Callaway E. (2021). Fast-spreading COVID variant can elude immune responses. Nature.

[15] Zeng B., Gao L., Zhou Q., Yu K., Sun F. (2022). Effectiveness of COVID-19 vaccines against SARS-CoV-2 variants of concern: A systematic review and meta-analysis. BMC Med..

[16] Chinese Center for Disease Control and Prevention (2020). Technical Guidelines for COVID-19 Laboratory Testing. China CDC Wkly..

[17] Niu P., Lu R., Zhao L., Wang H., Huang B., Ye F., Wang W., Tan W. (2020). Three Novel Real-Time RT-PCR Assays for Detection of COVID-19 Virus. China CDC Wkly..

[18] Daza-Torres M.L., García Y.E., Schmidt A.J., Pollock B.H., Sharpnack J., Nuño M. (2022). The impact of COVID-19 vaccination on California’s return to normalcy. PLoS ONE.

[19] Yang J., Zheng Y., Gou X., Pu K., Chen Z., Guo Q., Ji R., Wang H., Wang Y., Zhou Y. (2020). Prevalence of comorbidities in the novel Wuhan coronavirus (COVID-19) infection: A systematic review and meta-analysis. Int. J. Infect. Dis..

[20] Sun K., Chen J., Viboud C. (2020). Early epidemiological analysis of the coronavirus disease 2019 outbreak based on crowdsourced data: A population-level observational study. Lancet Digit. Health.

[21] Shim E., Tariq A., Choi W., Lee Y., Chowell G. (2020). Transmission potential and severity of COVID-19 in South Korea. Int. J. Infect. Dis..

[22] Ali M.R., Hasan M.A., Rahman M.S., Billah M., Karmakar S., Shimu A.S., Hossain M.F., Maruf M.M., Rahman M.S., Saju M.S. (2021). Clinical manifestations and socio-demographic status of COVID-19 patients during the second-wave of pandemic: A Bangladeshi experience. J. Infect. Public Health.

[23] Farshbafnadi M., Zonouzi S.K., Sabahi M., Dolatshahi M., Aarabi M.H. (2021). Aging & COVID-19 susceptibility, disease severity, and clinical outcomes: The role of entangled risk factors. Exp. Gerontol..

[24] Baden L.R., El Sahly H.M., Essink B., Kotloff K., Frey S., Novak R., Diemert D., Spector S.A., Rouphael N., Creech C.B. (2021). Efficacy and Safety of the mRNA-1273 SARS-CoV-2 Vaccine. N. Engl. J. Med..

[25] Dan J.M., Mateus J., Kato Y., Hastie K.M., Yu E.D., Faliti C.E., Grifoni A., Ramirez S.I., Haupt S., Frazier A. (2021). Immunological memory to SARS-CoV-2 assessed for up to 8 months after infection. Science.

[26] Bhuiyan T.R., Akhtar M., Akter A., Khaton F., Rahman S.I., Ferdous J., Nazneen A., Sumon S.A., Banik K.C., Bablu A.R. (2022). Seroprevalence of SARS-CoV-2 antibodies in Bangladesh related to novel coronavirus infection. IJID Reg..

[27] Vespa S., Simeone P., Catitti G., Buca D., De Bellis D., Pierdomenico L., Pieragostino D., Cicalini I., Del Boccio P., Natale L. (2022). SARS-CoV-2 and Immunity: Natural Infection Compared with Vaccination. Int. J. Mol. Sci..

[28] Pritchard E., Matthews P.C., Stoesser N., Eyre D.W., Gethings O., Vihta K.D., Jones J., House T., VanSteenHouse H., Bell I. (2021). Impact of vaccination on new SARS-CoV-2 infections in the United Kingdom. Nat. Med..

[29] Skowronski D.M., De Serres G. (2021). Safety and Efficacy of the BNT162b2 mRNA COVID-19 Vaccine. N. Engl. J. Med..

[30] Qian J., Zhao L., Ye R.Z., Li X.J., Liu Y.L. (2020). Age-dependent gender differences in COVID-19 in Mainland China: Comparative study. Clin. Infect. Dis..

[31] Peckham H., de Gruijter N.M., Raine C., Radziszewska A., Ciurtin C., Wedderburn L.R., Rosser E.C., Webb K., Deakin C.T. (2020). Male sex identified by global COVID-19 meta-analysis as a risk factor for death and ITU admission. Nat. Commun..

[32] Pradhan A., Olsson P.E. (2020). Sex differences in severity and mortality from COVID-19: Are males more vulnerable?. Biol. Sex Differ..

[33] Sharma G., Volgman A.S., Michos E.D. (2020). Sex differences in mortality from COVID-19 pandemic: Are men vulnerable and women protected?. Case Rep..

[34] Afroze F., Begum M.N., Ahmed T., El Arifeen S., Rahman M.Z., Rahman A.E., Mahfuz M., Kabir M.F., Kabir A., Amin R. (2024). Clinical characterisation, treatment outcomes, and case fatality risk of patients with different SARS-CoV-2 variants in Bangladesh. J. Glob. Health.

[35] Khan T.M., Afroz F., Mohiuddin G.M., Farzana A., Ashrafi F., Tangim S.F., Farzana F., Hossain E., Huda S.N., Paul S. (2022). Demographic Profile of COVID-19 Cases: Laboratory Experience in a Tertiary Care Hospital of Dhaka City. Sir Salimullah Med. Coll. J..

[36] Hossain M.I., Parvin S., Islam M.S., Alam M.J., Podder S., Datta R., Majumdar T.K., Hossain M.J., Ahmed F. (2021). Demographic profile and outcome of patients admitted to a COVID dedicated hospital in Bangladesh during the second wave. Medicine.

[37] Jubair M., Begum M.N., Rahman S., Haider S.M., Moon S.B., Hossain M.E., Rahman M.Z., Khan M.H., Alam A.N., Shirin T. (2023). SARS-CoV-2 Omicron variants in Bangladesh: Pandemic to endemic. Health Sci. Rep..

[38] Callaway E. (2021). Omicron likely to weaken COVID vaccine protection. Nature.

[39] Moore J.P., Offit P.A. (2021). SARS-CoV-2 Vaccines and the Growing Threat of Viral Variants. JAMA.

[40] Crotty S. (2021). Hybrid immunity. Science.

